# Manufacturing Technology and Mechanical Properties of Novel Pre-Impregnated Coatings as Applied to FRP “Sandwich” Composites

**DOI:** 10.3390/ma18204725

**Published:** 2025-10-15

**Authors:** Przemysław Golewski, Michał Budka

**Affiliations:** 1 Group of Solid State Mechanics, Department of Roads and Bridges, Faculty of Civil Engineering and Architecture, Lublin University of Technology, Nadbystrzycka 40 Str., 20-618 Lublin, Poland; 2 Laboratory of Civil Engineering, Faculty of Civil Engineering and Architecture, Lublin University of Technology, Nadbystrzycka 40 Str., 20-618 Lublin, Poland; m.budka@pollub.pl

**Keywords:** pre-impregnated coating, “sandwich” composite, digital image correlation (DIC), tensile test, adhesion test, three-point bending test

## Abstract

This article presents the manufacturing technology and mechanical properties of innovative pre-impregnated coatings (PCs). The base materials for PC are powders of metal oxides, non-metals, minerals and thermoplastic non-wovens. PC can be used in the manufacture of composites by methods such as vacuum infusion, autoclave curing or hand lamination. This is possible due to the novel PC structure consisting of a functional layer (FL) and a backing layer (BL). PCs are flexible so that they can be used on curved surfaces. In this work, five types of PC were subjected to a uniaxial tensile test. Depending on the powder used, failure force values ranging from 24.61 N to 28.73 N were obtained. In the next step, the pre-impregnated coatings were applied as a coating in “sandwich” composites made by vacuum infusion, which were subjected to three-point bending (3-PB) and adhesion tests. 3-PB tests proved that the coating remained integral with the substrate, even under high flexural deformation, while the adhesion achieved was in the range of 0.95 MPa to 1.57 MPa. PC can be used in many engineering products, e.g., for the coating of façade panels, roof tiles, automotive parts or rail vehicles, etc.

## 1. Introduction

Polymer matrix composites have been used for many years in such areas as space and aerospace [1], automotive [2] or marine [3,4]. Epoxy and polyester resins are most commonly used in composite products designed for civil engineering due to their favourable chemical, mechanical and technological properties. Epoxy resins are characterised by high mechanical strength, excellent adhesion to various materials, no shrinkage during curing, durability and resistance to ageing. In larger structures, e.g., bridge girders [5], façade panels [6] or building roofing [7], polyester resins are used, which are about 35% cheaper than epoxy resins. In addition, they are easy to process and have good chemical resistance. Regardless of the application, in addition to carrying the mechanical loads for which they are designed, these types of structures are also exposed to external exploitation loads such as UV radiation [8], elevated temperatures [9], erosion [10], impacts and scratches [11,12]. Solutions to this problem are various types of coatings, e.g., paints including those that intumesce when exposed to high temperatures [13,14,15]; rubber absorbers [16]; sol-gel [17]; gelcoats [18], which also give an aesthetic appearance; conductive nets [19]; ceramic mats [20,21]; and noncured graphene [22]. At present, however, there is no single universal method for surface protection of polymer composites, so research work is needed in this area.

Coatings of polymer composites can be applied either on one or both sides, as evidenced by a paper [23] in which Wang et al. used a polyurea coating to protect ultra-high-molecular-weight polyethylene (UHMWPE) laminates from ballistic impacts. Different options for the placement of the coating were considered: on the impact side, opposite the impact side and on both sides of the laminate. The study showed that most of the kinetic energy of the projectile is transferred through the polyurea coating when it is applied to the rear surface of the laminate.

One of the functions that coatings on GFRP composites can perform is anti-freeze properties. This type of effect can be achieved by applying a polyurethane coating, which is then textured by laser exposure and acquires superhydrophobic properties, making it difficult for an ice layer to build up [24]. Such a coating also has self-cleaning properties.

If the PMC composite has to work at elevated temperatures and, in addition, is to be exposed to impacts or scratches, then the best solution would be to use a TBC coating whose outer layer is yttria-stabilised zirconia (7YSZ). Obtaining such a coating on a PMC substrate is a challenging issue, as atmospheric plasma spraying technology (APS) is most often used to produce them. Hence, it is necessary to use either a suitable matrix or an interlayer. Semmler et al. [25] used a CFRP composite with a cyanoester matrix, the surface of which was sandblasted and then plasma-treated to improve wettability when building a TBC coating. Plasma spraying was also used in the work of [26], but in this case, it was an Al/FeCoNiCrMo coating on an epoxy matrix CFRP composite substrate. Surface preparation consisted of an acetone and sulphuric acid bath to remove the outer resin layer, followed by sandblasting and washing in an ultrasonic cleaner to eliminate any remaining broken fibres.

The dissipation of electrical charge from the surface, e.g., as a result of lightning strikes, can be an important function to be performed by the coating, especially when we are talking about aerospace structures. If the CFRP or GFRP composite does not have any protection, e.g., a metal mesh on the surface, then puncture of the fuselage surface and consequent catastrophe can occur. Hence, Das et al. [27] proposed a polyaniline-based coating. Before the coating was applied, the specimens were sandblasted to increase adhesion. Curing of the coating was performed in an oven at 130 °C. The study determined that the minimum thickness should be 345 μm.

Cilento et al. [28] emphasise that polymer composites have poor properties considering the effect of heat flux, even in the range of 300–400 °C. The organic surface degrades, producing smoke and toxic compounds [29]; hence, a TBC coating made of carbon nanotubes has been proposed. It is optimal to make two layers of coating, which allows for lowering the temperature of the heated CFRP laminate and also maintains a higher elastic modulus after the thermal impact test.

Nanotechnology was also used in a paper [30] to produce a heat-resistant coating. The authors produced an original prepreg consisting of poly(vinyl alcohol) (PVA) containing ammonium polyphosphate (APP) and sepiolite nanofillers (SP), and the hole was placed on a glass fabric support layer. This finished product was then laminated as a coating onto the GFRP composite and allowed the back wall temperature to be reduced during flame exposure.

One method of protecting composite surfaces and, at the same time, improving their aesthetics is gelcoats. Their advantages include the ability to cover large surfaces (e.g., boats [31,32]), continuity of the coating structure, protection against damage and atmospheric effects. Disadvantages, on the other hand, are difficulty in controlling the application, the need for curing, visible surface defects such as streaking or porosity and thus repair problems. Despite this, scientific work is currently being carried out on gelcoats, but their composition is modified by the addition of nanoparticles. Their size is below 100 nm, and both metals and ceramics can be used. Nanoparticles, due to their large developed surface area, interact with the polymer matrix, and even a small addition (0.5–6% by volume) causes large changes in the strength and stiffness of the coating [33,34]. Depending on the filler material, gelcoats can also have fire-retardant properties. Pomázi et al. [35] made epoxy resin-based gelcoats with 5%, 10% and 15% ammonium polyphosphate (APP) content and compared their thermal stability, glass transition temperature, enthalpy of crosslinking reaction and fire resistance with non-flammable and commercial gelcoats. Considering the rate of heat release, a similar behaviour for 10% APP was observed, similar to that of the commercial gelcoat.

A separate important group consists of layers and coatings made of macro and microparticles, the matrix of which is often epoxy resin. Powders of both metals, e.g., copper [36] and non-metals, e.g., in the form of quartz sand [37], alumina [38], mineral fillers [39] and zirconium oxide [40], are used. As mentioned above, they can form the final coating and protect the PMC composite substrate from mechanical or thermal stresses. Their second application is to provide an interlayer role, e.g., for coatings produced by flame spraying [41]. The presented technologies in the field of powder application share a common feature. They are produced on an existing composite product, so this is an additional process that can be difficult to perform when the surface is curved, e.g., airfoils or automotive parts. Other solutions are therefore needed, such as in the presented article, where the coating is in the form of an innovative prepreg.

In summary, the subject of coatings on PMC composites is very broad and constantly evolving, as evidenced by the wide variety of applications, substrate types and surface preparation methods. In most cases, the authors apply a coating to a fabricated composite product. This approach may require the use of, for example, sand blasting prior to the application of the coating, which affects the structure of the composite and thus may reduce its strength.

**Figure 1:**
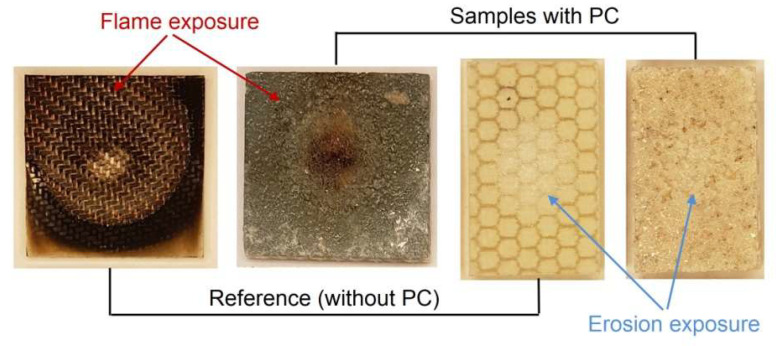
PC as multifunctional coatings.

Therefore, in this work, the authors have proposed an original technology for the application of ready-made PC coatings, which can be used in the manufacture of composites by a number of methods, e.g., vacuum infusion, hand lamination or autoclave curing. The main objective of the work was to develop production equipment and perform mechanical tests on the manufactured PC and “sandwich” composites with PC. The proposed coatings are flexible, which allows them to be applied to curved surfaces and can be cut to size or fabricated to a specific shape. The pre-impregnated coatings are multipurpose, as different powders can be used to achieve different functional (e.g., flame or erosion protection, Figure 1) or decorative effects. The technology presented here is a continuation of the work described in article [42], where manual spreading of the powder layer on the surface of the CFRP prepreg was used, and the whole product was cured in an autoclave.

## 2. Materials and Methods

The main materials for the PC presented in this article are powders, oxides and minerals, as well as a thermoplastic non-woven copolyester with a melting point in the range of 138–148°C and a grammage of 84 g/m^2^. This plays the role of a matrix. The PC consists of the BL and the FL, which are bonded together by welding on a thermo-transfer press. The following powders have been used for the FL:

1. Silicon carbide green;
2. Glauconite;
3. Stainless-steel shot;
4. Quartz sand;
5. Chrome electro-corundum.

**Figure 2:**
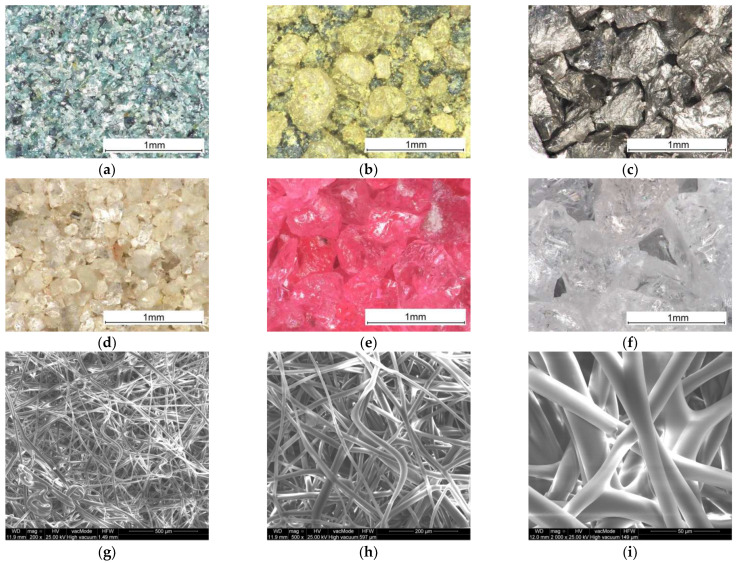
Microscope images of the powders used (×150) and the thermoplastic non-woven fabric (×200, ×500, ×2000): (**a**) silicon carbide green; (**b**) glauconite; (**c**) stainless-steel shot; (**d**) quartz sand; (**e**) chromium electro-corundum; (**f**) white electro-corundum; (**g**–**i**) thermoplastic non-woven fabric.

The grain size parameters are given in Table 1. It should be mentioned that the given order will be retained in each of the three types of strength tests as the batch number. White electro-corundum was used to create the BL in batches 1–5. Digital microscope images of the powders used and SEM microscope images for the thermoplastic non-woven are shown in Figure 2.

Powders 1, 3, and 4 and white electro-corundum were sourced from the “Rewa” company (Nowy Redzeń, Poland). Glauconite is a natural mineral and comes from the Lublin area (Poland), as does the quartz sand used. The thermoplastic non-woven fabric was produced by ”TMBK Partners” (Warsaw, Poland).

**Figure 3:**
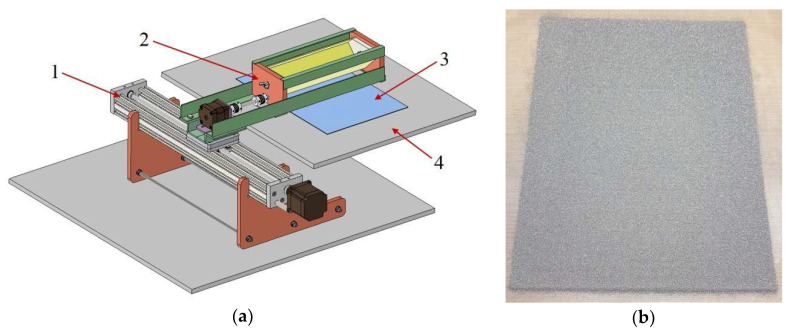
(**a**) Schematic of the powder layer spreading device (1. linear drive; 2. powder feeder; 3. formed powder layer; 4. base of the thermo-transfer press); (**b**) formed powder layer.

This wide variety of powders used in terms of grain size, type and colour was chosen deliberately for three key reasons. The first, and most important, is to test the capabilities of the new original device shown in Figure 3a. The device was designed to be universal in terms of a wide range of grain sizes. The second reason is to test whether the thermoplastic non-woven copolyester, the grammage of which was selected as a result of technological trials, would be able to be used independently of the type of powder material. The third reason, which is significant from an aesthetic point of view, is the final colour effect of the resulting prepreg, which is planned for use on the surface of, for example, façade composite panels.

### 2.1. Manufacturing Technology for Pre-Impregnated Coatings

The pre-impregnated coatings presented in this paper are a new product, so the technology developed is described extensively, along with the results obtained. The technology for the production of multifunctional coatings can be carried out manually, semi-automatically or on an automated production line.

The simplest method is the manual method, which involves manually spreading a layer of powder on the surface, with the advantage of using a framing tool to achieve a constant thickness, as shown in the work of [42]. However, the method is time-consuming and labour-intensive to apply, especially for low-gradation powders of the range of 50 micrometres and large areas such as 0.5 m × 1.0 m. It can only be used for unit production and for flat products.

The construction of an automated process line is only justified if we will be producing significant quantities of pre-impregnate at an industrial level. Therefore, a semi-automatic technology was developed for laboratory use. An original device was constructed to automatically spread the powder layer (Figure 3a), while other operations, such as laying down the non-wovens, were performed manually.

The construction of the device uses a linear drive, (1) ”V-Slot”, with a transversely attached arm, on which the powder feeder (2) is placed. This makes it possible to spread an even layer of powder (3) in a controlled manner directly on the table of the heat transfer press (4), as can be seen in Figure 3b. It should be mentioned that the feeders (2) are interchangeable and also have a continuously adjustable gap through which the powder is fed, which finally allows the use of powders of different gradations.

The dispersion speed of the powder layer can be adjusted in two ways:

- By adjusting the rotational speed of the powder feeder roller while maintaining the same number of passes;
- By adjusting the number of passes while maintaining the same rotational speed of the feeder roller.

**Figure 4:**
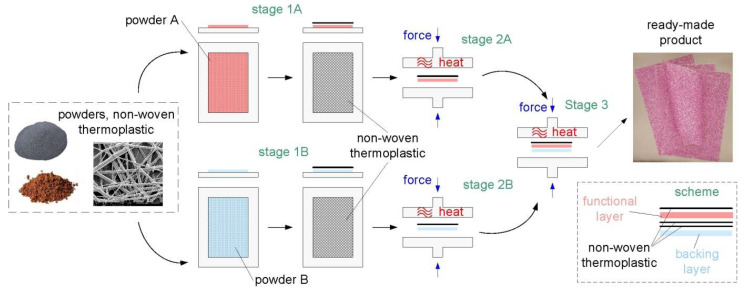
Schematic diagram of PC manufacturing.

In this study, PC manufacturing technology took place in several stages and is shown in Figure 4.

The first stage consists of spreading a layer of powder on a flat surface (Figure 3b), laying down the thermoplastic non-woven fabric and then welding under pressure with a REV 3S thermo-transfer press from ”Transmatic” (Lazzate, Italy) equipped with digital control of both temperature value and welding time.

**Figure A1:**
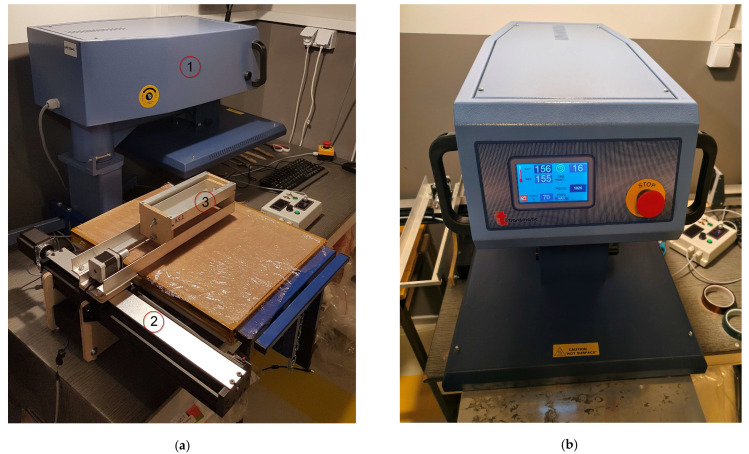
Equipment for the production of prepregs: (**a**) view during the opening of the thermal transfer press (1. Press; 2. linear drive; 3. powder feeder); (**b**) view during welding.

The PCs presented consist of two layers—the FL and the BL—which is why there are two processes, A and B, in Figure 4. The third and final process is to join the two previously manufactured layers using an additional thermoplastic non-woven. The welding time and temperature in stages 2A, 2B and 3 were 300 seconds and 155 °C, respectively. Figure 4 shows an example of a finished PC product made using chrome electro-corundum powder. Photographs of the devices used are included in the Appendix (Figure A1).

The weakness of this technology is that the press used in this study has a table size of 40 × 50 cm, while some composite products, especially in the field of civil engineering, can have overall dimensions of several metres. For example, the PC coating of a façade panel measuring 1.2 m × 1.0 m would have to consist of 6 PC sheets. The solution to this problem could be to use larger presses or to use a continuous welding line. In this case, only the width would be a limitation. Of course, individual PC sheets can be stacked next to each other. In this case, however, it would be necessary to solve the problem of discontinuities at the joining point. It is also possible to custom cut any shape and combine it into a larger area to create different geometric and colour patterns.

**Figure 5:**
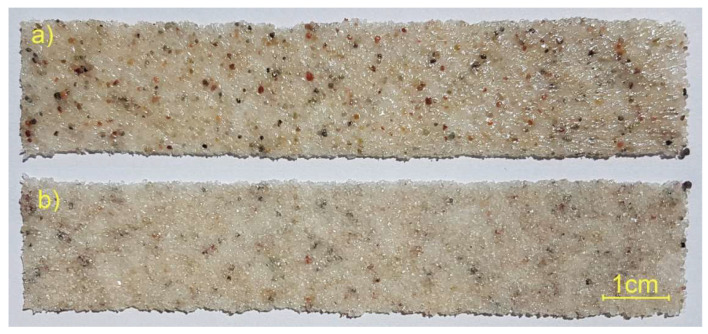
PC fragment: (**a**) view from the FL; (**b**) view from the BL.

The advantages of this technology are the relatively low price of the semifinished products, the repeatability of dimensions and grammage and the wide range of powders used. However, for the range of powder weights of the BL and the FL, an appropriate thermoplastic non-woven grammage must be selected so that the FL is tightly covered with the polymer material. The grains in the BL side are not completely saturated with the thermoplastic material (Figure 5). This means that the grains on the BL side are only partially bonded to each other, and voids are created, which provide space for the liquid resin introduced at the composite manufacturing stage.

In this case, in addition to the adhesion between the grains and the resin, mechanical joints are formed, which makes the coating formed durable and prevents delamination during bending.

When spreading powder from the feeder, defects can occur, such as the following:

- One-sided cavities;
- Uniform defects covering the entire sheet;
- Excessive powder at the beginning and end of the layer, resulting from the delayed movement of the feeder.

**Figure 6:**
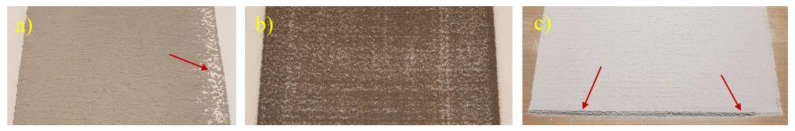
Defects during the spreading of the powder layer: (**a**) one-sided cavities; (**b**) uniform defects covering the entire sheet; (**c**) excessive powder at the beginning and end of the layer, resulting from the delayed movement of the feeder.

These disadvantages are shown in Figure 6; hence, in many cases, the layer of distributed powder must be of sufficient thickness to evenly cover the entire surface. This involves spreading excess powder, which remains unused after welding and is returned to the next cycle.

The obtained PC grammages can be within a wide range of values, which depends, e.g., on the powder material used, the grain size or the number of layers (Table 2). In the table, there is also a batch 6 made only of copolyester (3 layers with a grammage of 84 g/m^2^), which can be considered as a reference, as the amount of thermoplastic material is the same as in batches 1 to 5.

The thickness of the PC, shown in Table 2, has been calculated as the average of 11 measurements along the length of the specimens at a distance of 250 mm. The PC is a new product; therefore, it is important to comment on the thickness deviations obtained. If the results are expressed as a percentage, then the range of thickness deviations for the PC obtained is in the range of 3.73% to 7.02%. This type of material has no equivalent in the literature; however, considering epoxy–carbon prepregs, as in the work of [43], then a range of 0.99% to 3.96% was obtained. It should be noted that the new PCs presented in this work are based on grains that are not themselves regular and, for each grain size, fall within a certain size range. In contrast, the epoxy–carbon prepregs only have reinforcement fibres and a polymer matrix. Hence, the presented range of percentage deviations from the average thickness should be considered very good, as the lower value of 3.73% is close to the upper range of 3.93% for professional prepregs.

**Figure 7:**
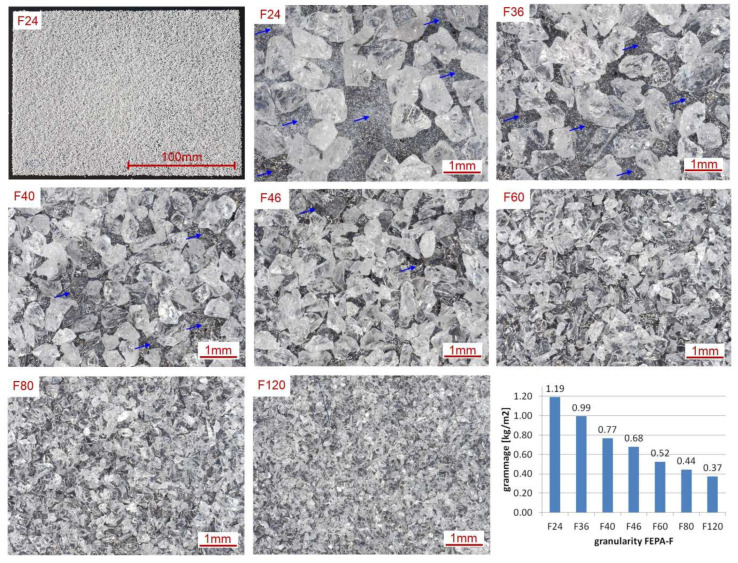
View of the backing layer for different grain sizes of electro-corundum.

The grammage of the PC also depends on the granularity of the BL powder, which, as mentioned, is supposed to join with the liquid resin. In this study, welding process tests were carried out for 7 BL formats of 150 mm × 210 mm to determine the grammage and to make microscopic observations (Figure 7).

The manufactured sheets represent stage 2B, as shown in Figure 4. The grammage of the BL decreases as the grain size of the electro-corundum powders used decreases and is in the range of 1.19 kg/m^2^ to 0.37 kg/m^2^. As the manufacturing of the PCs was performed for the first time, the highest grain size of F24 (0.745 mm) alumina was used for their fabrication. However, if we analyse the microscopic images in Figure 7, the phenomenon of incomplete coverage of the thermoplastic non-woven by the grains can be seen. The arrows indicate the more extensive areas where copolyester is visible.

The limiting grain size is F46, where the entire surface is covered with grains. The use of small gradations, such as F120, may result in complete supersaturation of the grains at stage 3, i.e., welding to the FL. Hence, adhesion and bending tests will also be carried out for other gradations of the BL than F24 in further studies.

### 2.2. Vacuum Infusion Technology

**Figure 8:**
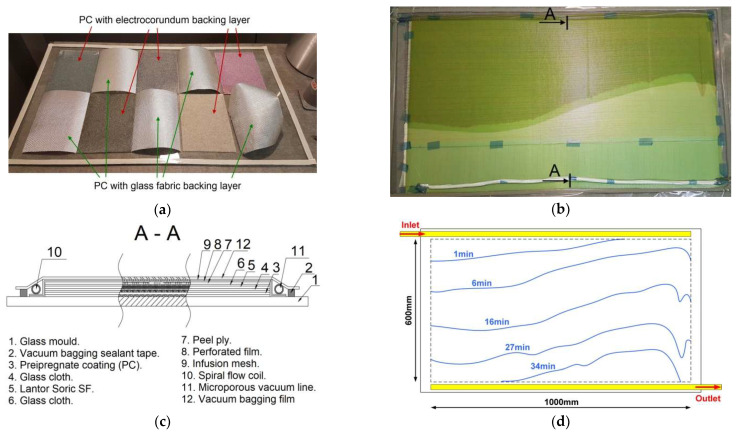
Vacuum infusion process: (**a**) PC placement on glass plate; (**b**) resin flow for 16 min; (**c**) view of layers; (**d**) time course of infusion.

**Figure A2:**
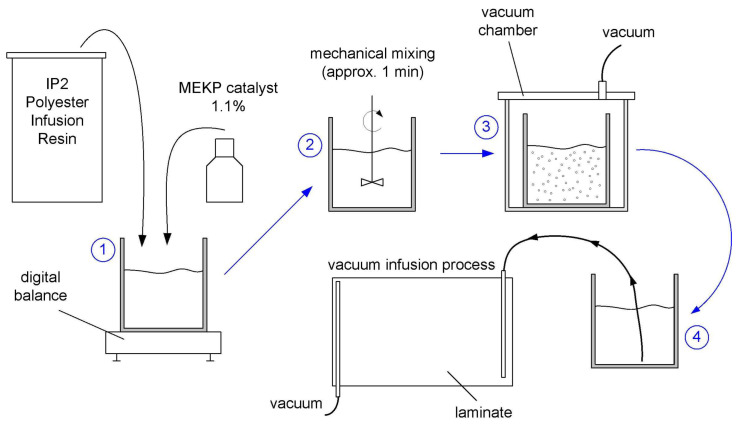
Process of preparing a mixture of resin and catalyst for vacuum infusion.

The PC “sandwich” composite for adhesion and bending tests was made by vacuum infusion (Figure 8). "Soric SF" with a thickness of 3 mm from "Lantor" (Veenendaal, The Netherlands) was used as the core, while glass twill fabric with a weight of 280 g/m^2^ was used for the double-sided laminates. The arrangement of the layers was 3 x 0°/core/3 x 0°. IP2 polyester infusion resin was used along with MEKP catalyst (Easy Composites, Stoke-on-Trent, UK). MEKP catalyst is a solution of methyl ethyl ketone peroxide and cumyl hydroperoxide in a phlegmatiser. The addition of the catalyst was 1.1% by weight of the resin. The vacuum infusion process proceeded as follows:

- Preparing the glass plate by coating it with a thin layer of wax;
- Arrangement of the PC sheets so that the FL was on the glass side (Figure 8a);
- Placement of three layers of reinforcement involved a “Soric SF” core; again three layers of reinforcement; and also peel-ply, perforated film and infusion mesh. The package was closed with a vacuum bag, in which a vacuum of 300 hPa was applied;
- Preparation of the resin/catalyst mixture by mechanical mixing, placing the container with the mixture in a vacuum chamber to remove the air. This process is illustrated in Figure A2 in the Appendix.

A schematic of the progression of resin inflow into the bag can be seen in Figure 8d, together with the marked lines showing the time to reach the filling level. The resin inflow was via a spiral tube with an outer diameter of 4.5 mm, while the air was discharged via an ‘MTI’ microporous hose from ‘DD-Compound’ (Ibbenbüren, Germany). In addition, Figure 8b shows the actual fill level of the vacuum bag for 16 minutes after the valve was opened.

Figure 8a shows 10 PC sheets with an electro-corundum white backing layer and glass fabric. In this study, only the results for the PC with the white electro-corundum backing layer will be discussed.

**Figure 9:**
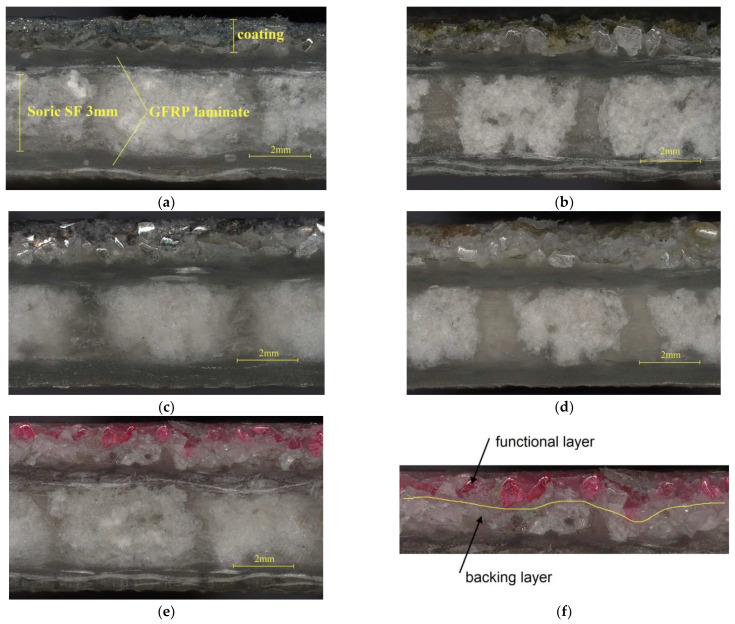
Cross-sections of the composites: (**a**) silicon carbide FL; (**b**) glauconite FL; (**c**) steel shot FL; (**d**) quartz sand FL; (**e**) chromium electro-corundum FL; (**f**) division of PC into BL and FL.

For the “sandwich” composites, observations were made on a Keyence VHX-970F (Osaka, Japan) microscope, which allowed the thickness of both the coatings and the entire composite to be measured (Figure 9).

In Figure 9a, the cross-section is highlighted:

- A 3 mm thick "Soric SF" core;
- GFRP laminate layers on both sides;
- Single-sided PC coating.

In Figure 9f, due to the colour difference, the white electro-corundum BL, which bonds to the resin during infusion, and the FL (pink colour), which is visible from the outside of the composite, can be easily distinguished. The resulting coatings are consistent with no voids or cracks. Furthermore, their thickness is uniform along the entire length of the composite. This is due to the fact that thermoplastic non-woven fabric has a uniform thickness and appropriate grammage, and during its melting in processes 2A and 2B, as shown in Figure 4, only one layer of powder grains can be bonded. Table 3 collects the average results of the thickness measurements of the composites taken at 15 points, together with the specified deviation. Although the thickness deviation is larger than for the reference specimens without PC, it is still at a low level in the range of 0.05 mm to 0.09 mm. For most applications, such a deviation is visually imperceptible and does not adversely affect the properties of the product.

### 2.3. Strength Tests

**Figure 10:**
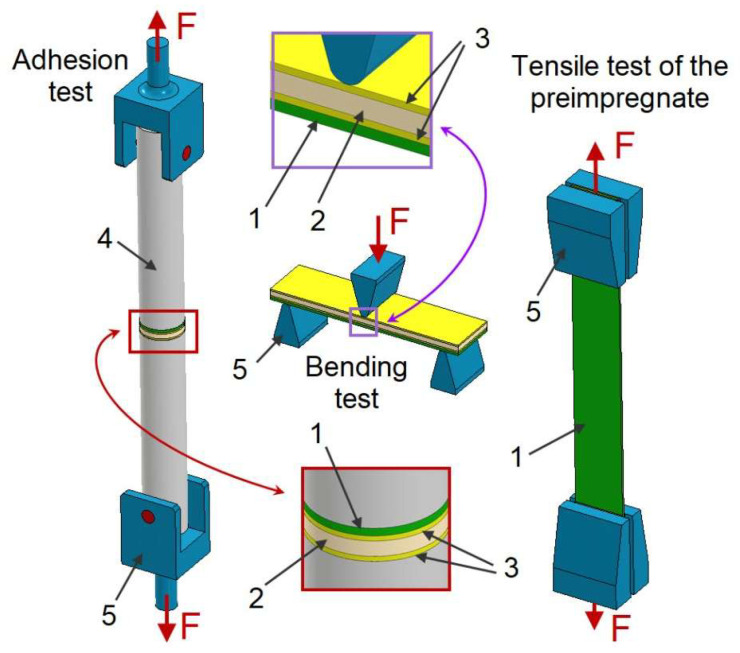
Strength test diagrams: 1. pre-impregnated coating; 2. core; 3. GFRP laminate; 4. aluminium bars; 5. holders and supports.

**Figure 11:**
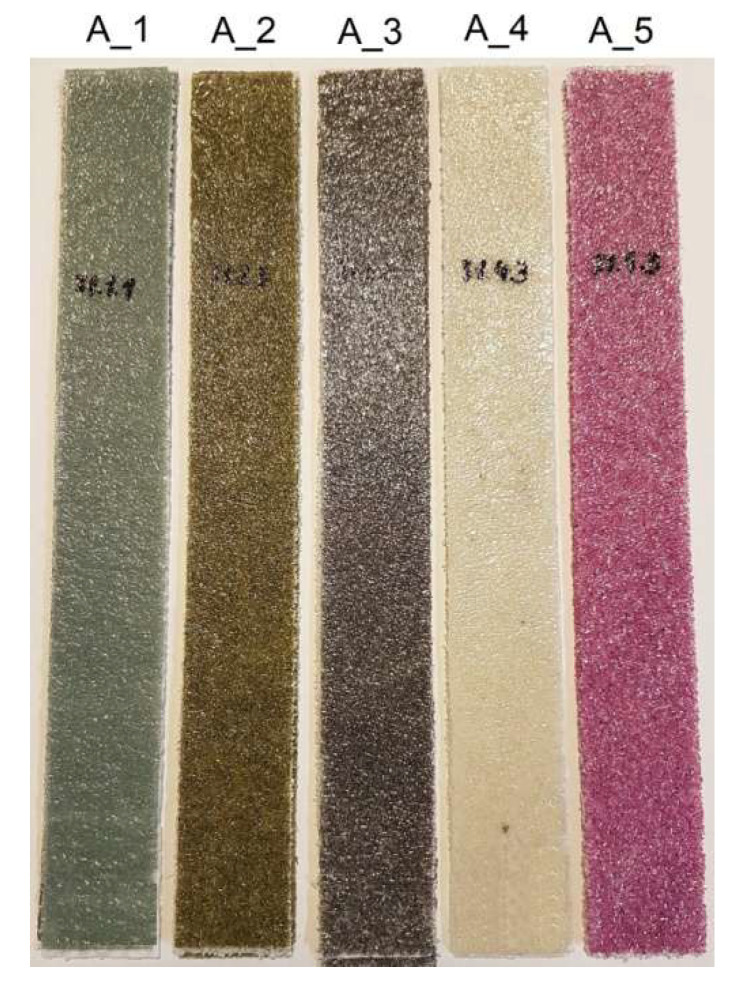
PC specimens for uniaxial tensile tests.

Three types of strength tests were carried out, schematics of which can be seen in Figure 10:

- Pre-impregnate tensile for 30 mm × 250 mm specimens (Figure 11) measuring 30 mm × 250 mm—batch A (three specimens for each batch);
- Bending for PC “sandwich” composite—30 mm × 120 mm specimens—batch B (three specimens for each batch);
- Adhesion for the “sandwich” composite with PC—25 mm diameter circular specimens—batch C (five specimens for each batch).

The “sandwich” composite specimens were cut on an ATM Solutions CNC plotter (Łomianki, Poland) using a 3 mm diameter carbide cutter.

Adhesion tests required additional parts made from 2017A-grade aluminium rods that were 25 mm in diameter and 125 mm long. The ends of the rods were sandblasted with F40 grit electro-corundum before bonding. 3M Scotch-Weld 2216 B/A epoxy adhesive was used to make the joints.

**Figure A3:**
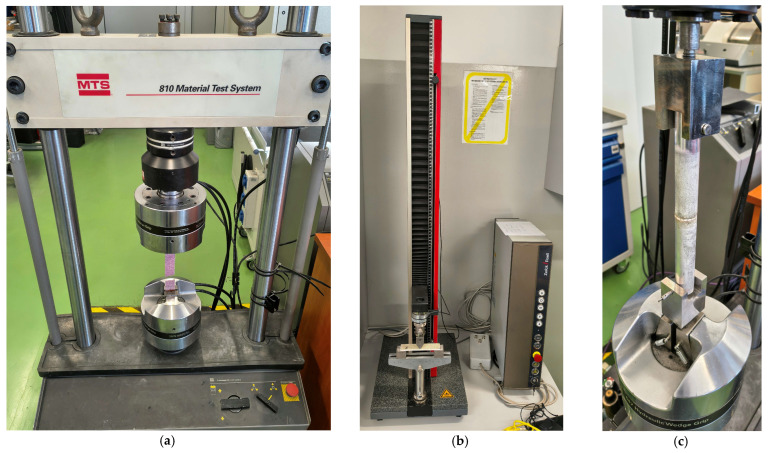
Photographs obtained during strength tests: (**a**) uniaxial tensile test of pre-impregnated coatings; (**b**) 3-PB test; (**c**) adhesion test.

Tensile tests of the PC and 3-PB tests were carried out on a Zwick/Roell testing machine with a range of up to 2.5 kN, and adhesion tests were carried out on an MTS 100 kN machine. The tests were conducted statically with a constant displacement of 1 mm/min. Photographs of the specimens mounted on the strength testing machines are shown in Figure A3 in the Appendix. A digital image correlation system, “Aramis” (Carl Zeiss, Oberkochen, Deutschland GmbH), was also used in the tests to observe deformations and determine elastic constants for the PC. The force–displacement graphs were performed using Diadem 2019 software.

In terms of statistical analysis, arithmetic means, standard deviation and *t*-test for difference of two means in paired specimens were determined in Excel.

## 3. Results and Discussion

The new PC and PC “sandwich” composite products required laboratory testing. The tests were divided into three stages:

- PC tensile strength test with different powders for the FL;
- Adhesion tests of the “sandwich” composite with PC;
- Three-point bending tests of the “sandwich” composite with PC.

### 3.1. PC Tensile Tests

PC uniaxial tensile tests were carried out for five batches (Figure 11), with three specimens in each batch. The sixth reference batch consisted of specimens of melted non-woven fabric in an identical amount to that used in the PC. Overlaps of 30 mm × 50 mm were bonded to the ends of the PC to reduce the impact of the jaws of the testing machine grips. Knowledge of the tensile strength of this new material is needed for several technological reasons:

- When PC is manufactured in tape form and must be wound onto a spool. The tension force of the spool must not cause plastic deformation of the tape;
- PC is applied to the product, and the tension force must not exceed the limit for the formation of permanent deformations.

**Figure 12:**
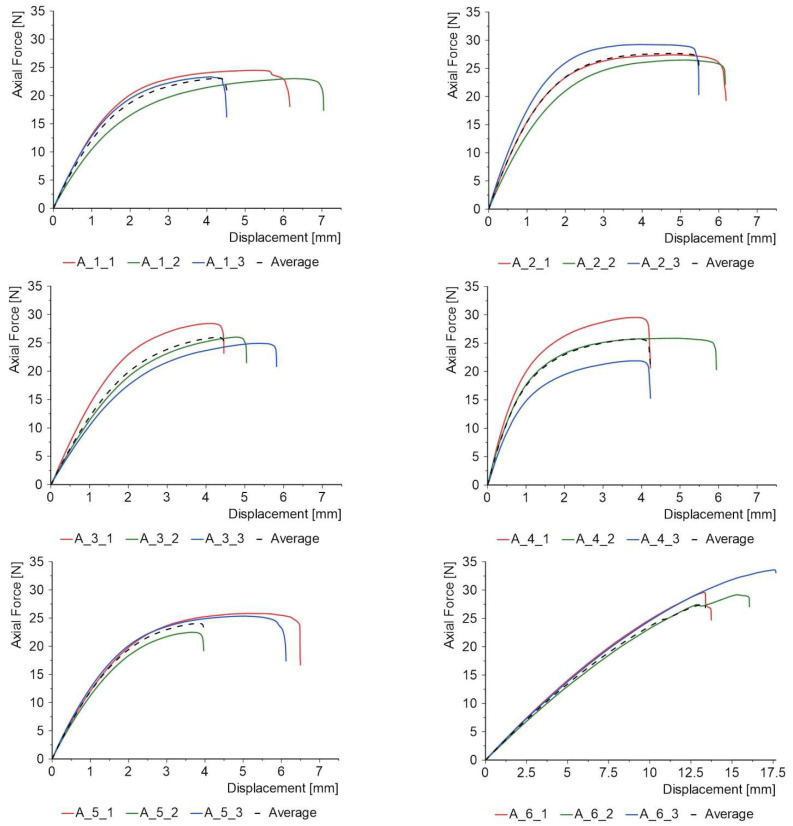
Force–displacement diagrams of uniaxial tensile PC specimens.

Figure 12 summarises the results of the uniaxial tensile tests in the form of force–displacement diagrams. These are characterised by an initial linear range, after which the graph curves until failure. An exception is the reference specimens made only of melted thermoplastic non-woven fabric (batch 6). In this case, the graph does not curve significantly, and the specimens behave linearly until failure.

In the graphs shown in Figure 12, the average result for each batch is shown. Each curve can be approximated by a polynomial function in tandem with the general Equation (1):

(1) $$y=a_{1}x+a_{2}x^{2}+a_{3}x^{3}+a_{4}x^{4}$$

where

*x* denotes the displacement [mm].

*y* denotes the axial force value [kN].

In the above equation, the parameters for each curve indicated by the dashed line were chosen to obtain a high coefficient of determination and are collected in Table 4.

The most relevant are the first two coefficients: a_1_ and a_2_. Coefficient a_1_ is responsible for the initial stiffness of the material and the linear response. This is the range in which there is proportionality between force and displacement. The highest value was obtained for a material in which the FL was made of quartz sand, while the lowest value was obtained for the FL made of steel shot. The second coefficient (a_2_) is related to the non-linear behaviour of the material when the proportional limit is exceeded. A negative value of a_2_ indicates that the material is softening, while a positive value indicates hardening [44]. In the materials tested, each of the a_2_ coefficients is negative, which is a common feature and indicates the initiation of the material's failure process. The last two coefficients (a_3_ and a_4),_ reflecting more complex, higher-order non-linear effects in the material's behaviour, may indicate yielding of the product just prior to failure.

**Figure 13:**
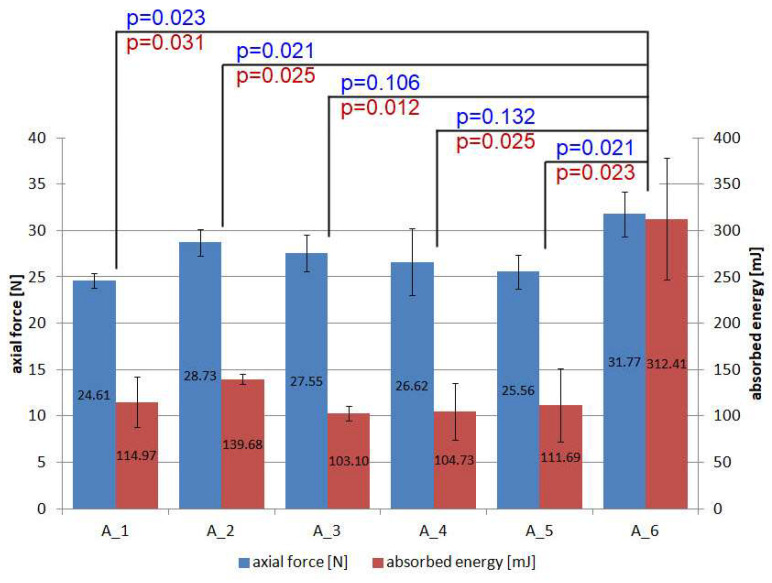
Maximum forces and absorbed energy of PC specimens during tensile test.

Based on the above data, the results were compiled in the form of a graph (Figure 13) showing the mean values for the maximum forces and the absorbed energy (E), calculated as the area under the force–displacement diagrams (Equation (2)). The standard deviation is also shown for each batch.

(2) $$E=\int_{x_{1}}^{x_{2}}F\left(x\right)\cdot dx$$

where

F(*x*) denotes the force–displacement relationship [N].

*x*_1_ and *x*_2_ denote the initial and final displacement [mm].

The highest parameters are achieved by reference specimens made of three layers of non-woven thermoplastic material, bearing in mind that the same amount of this material is provided in each remaining batch. Thus, creating a structure with powders from different materials and with different gradations reduces the strength of the thermoplastic material by between 22.54% and 9.57%. However, this should not be seen as a disadvantage, as the aim of the non-woven thermoplastic is to combine the individual powder layers into a single product. This is carried out so as to achieve uniform thickness and strength sufficient for manual manipulation during composite manufacture while maintaining flexibility. This objective was reached. Further increases in strength can be achieved by additionally reinforcing the thermoplastic non-woven with, for example, dispersed fibres or nano-additives [30]. There are statistically significant differences (*p* < 0.05) when comparing reference batch A6 with A1, A2 and A5.

Much greater differences occur when the absorbed energy is considered. Its value is significant and is related to the deformation of the material to failure. The decrease in this parameter in relation to the reference specimens is in the range of 55.29% to 67.00%. Statistically significant differences (*p* < 0.05) exist for all batches compared to the reference batch.

**Figure 14:**
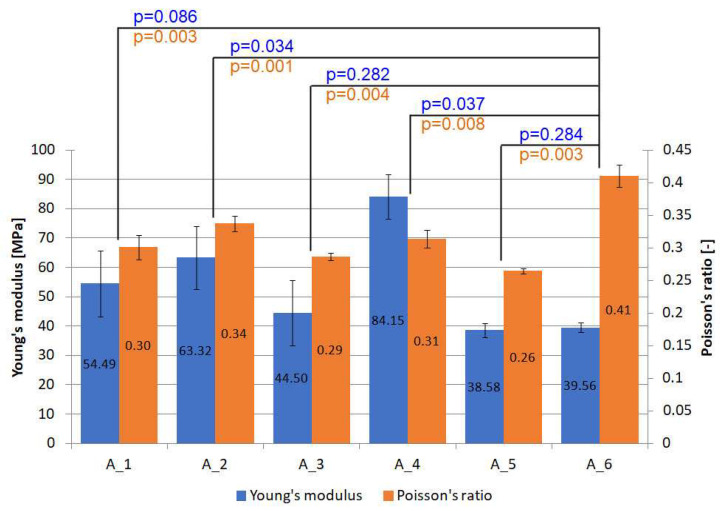
Young’s modulus and Poisson’s ratio for PC specimens.

An important parameter from the point of view of PC numerical modelling is Young's modulus and Poisson's ratio (Figure 14). These parameters were calculated from strain observations using the Aramis system. The Young's modulus for each PC shows an increase compared to the reference batch. The maximum value was reached for the quartz sand specimens (batch 4), and this was an increase of 112.71%. The module of elasticity obtained is three orders of magnitude lower than that for the GFRP laminate [45]. Therefore, this type of product can be easily adapted to a curved surface, even before it is impregnated and will work elastically even under significant deformation of the composite substrate without fracturing. There are statistically significant differences (*p* < 0.05) when comparing reference batch A6 with A2 and A4.

The second important parameter is the ratio of transverse to longitudinal strain—Poisson’s ratio. In this case, it is reduced relative to the reference batch (0.41) to a range of 0.26 to 0.34. This range is close to the values achieved by GFRP laminates [46] or steel. This is a favourable change compared to pure thermoplastic material, since the difference in strains of the substrate material and PC can result in additional stresses and consequent delamination or cracking of the PC. Statistically significant differences (*p* < 0.05) exist for all batches compared to the reference batch.

**Figure 15:**
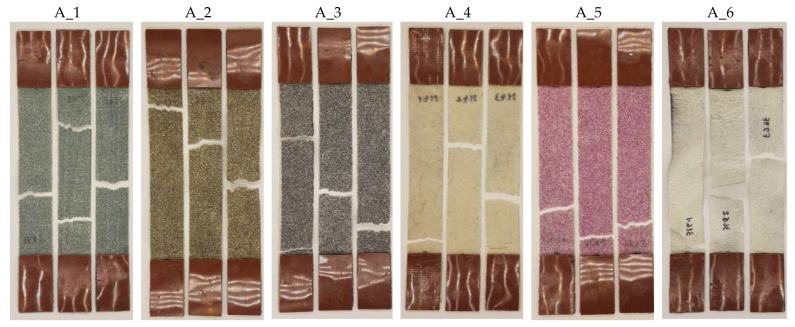
Photographs of PC specimens after failure.

Figure 15 summarises the images of all PC specimens and the reference batch after the tensile tests. In almost every case, there is only one crack in the specimen at different distances from the overlap. In general, the cracks progress perpendicular to the line of action of the load. The small “jaggedness” is due to the heterogeneity of the powder layers, in which the grains themselves are not regular, their distribution is random, and the gradation of the powders is within a certain range, resulting from screening on the sieves.

**Figure 16:**
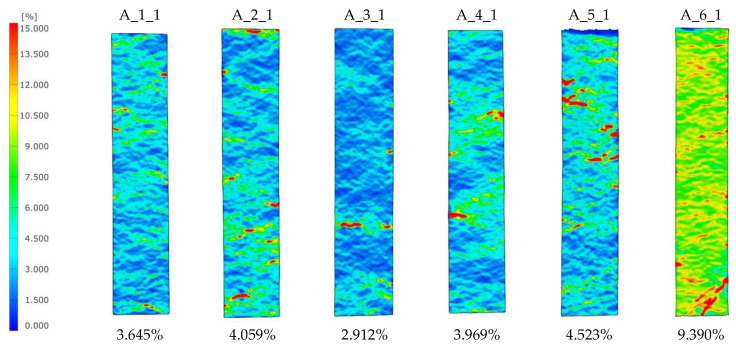
Principal strains (I) just before failure of the PC specimens.

The locations where PC failure occurs can also be determined using the Aramis system. Figure 16 presents the principal strain (I) maps for one specimen in the batch just before fracture initiation. The figure also shows the mean value of the visible strain field. Most often, the cracking process starts from the lateral edge, but the concentrations themselves also appear inside the specimen at some distance from the edge. Despite the fact that the specimens were manufactured from the beginning in the 30 mm dimension (they were not cut to size), the strain fields do not show any negative effects at the edges themselves that would indicate, for example, a lower grammage; this is particularly evident throughout the range, as well as for smaller strains. This effect is a good indication of a well-made product that does not require additional processing.

### 3.2. PC Adhesion Tests

Adhesion tests were carried out to determine the stresses that cause the coating to detach from the substrate and to determine the failure model. This type of test is more difficult to perform due to the precision of the machining of the intermediate components—in this case, aluminium rods with a diameter of 25 mm and a length of 125 mm—the bonding process and the assembly on the testing machine.

**Figure 17:**
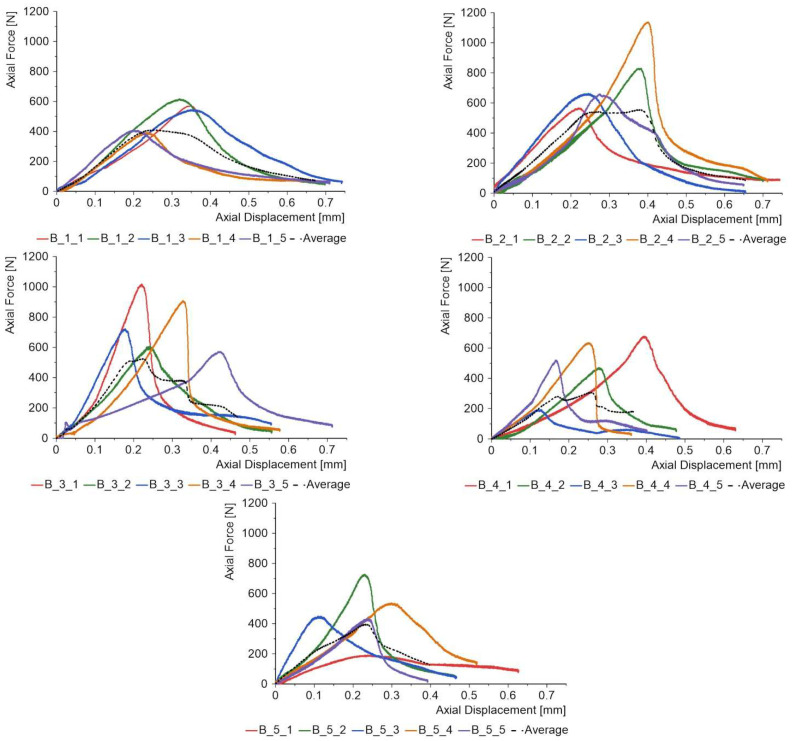
PC specimens’ adhesion test results.

Figure 17 summarises all the graphs for the five batches. The graphs are, in most cases, characterised by a symmetrical shape, which shows that the coating damage process does not occur rapidly as it did during the PC tensile tests.

**Figure 18:**
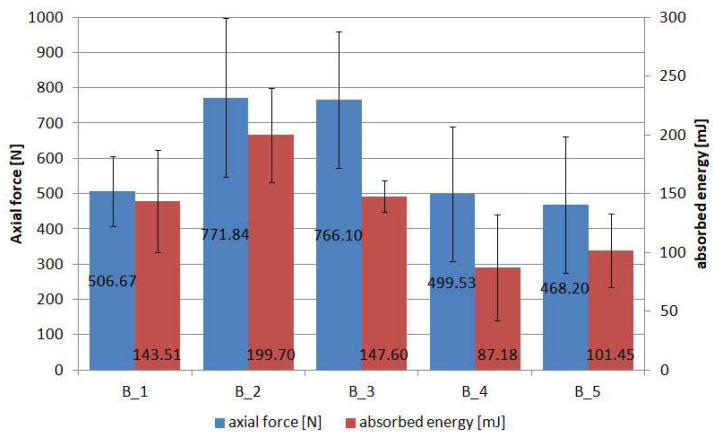
Average values of maximum forces and absorbed energy during adhesion tests of PC specimens.

Figure 18 presents the average values of maximum forces and absorbed energy. Note the values for the relative percentage standard deviation, which reflect the poorer repeatability of the results than for the previously presented batch A. Considering the maximum forces, the %RSD is in the range of 19.74% (batch 1) to 41.35% (batch 5), while for the absorbed energy, it is 9.00% (batch 3) to 51.45% (batch 4).

Analysing the literature, in [47], gelcoat coatings were produced on PMC composites with an adhesion of 4 MPa with a standard deviation of 2.4 MPa and a %RSD as high as 60%. Much higher adhesion can be achieved with coatings made by cold spray [48]. In this case, the adhesion depends on the number of passes of the sprayed nozzle over the material and, therefore, the thickness of the coating and is at 6.3 MPa with a standard deviation of 0.12 MPa and a %RSD of 1.9%.

**Figure 19:**
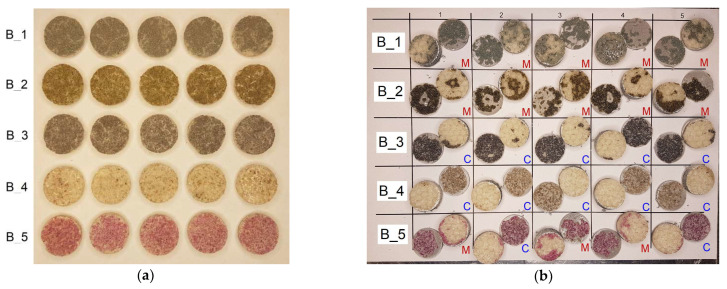
Adhesion test: (**a**) view of specimens before test; (**b**) view of specimens after adhesion tests (C—cohesive failure; M—mixed failure).

The poorer repeatability of the results in the tests carried out may have been due to the fact that the specimens were cut on a plotter by milling. This causes the edges of the PC to fray as the powder grains are pulled out rather than cut through. The imperfection in the manufacture of the specimens also comes from the process of bonding the sample to the aluminium parts. In this case, the specimens were bonded using a suitable jig to ensure alignment, and the adhesive was applied to all four surfaces; after which, the specimens were pressed down by hand and allowed to cure the adhesive. A final important factor that can negatively affect the repeatability of results is the way the specimens are fixed in the testing machine. In this study, two hinges were used to eliminate a possible eccentricity. Therefore, five specimens per batch were used in the adhesion tests. Complex failure processes take place in this type of specimen, the end results of which can be seen in Figure 19. Figure 19a shows the sets of specimens before bonding to the aluminium rod. After the tests, failure sections of the specimens were cut off, allowing them to be fully assembled in Figure 19b.

The most common predominant failure is between the BL and the FL, which can be classified as cohesive. This is understandable, as the two different powder gradations are bonded together with a melted copolyester fabric, which has a tensile strength of 3.65 MPa, while the 3M Scotch-Weld 2216 B/A epoxy adhesive strength claimed by the manufacturer is between 20 MPa and 50 MPa. Cohesive failure predominates mainly in groups B_3, B_4 and partly B_5. In contrast, in groups B_1, B_2 and partly B_5, a mixed cohesive–adhesive failure model dominates, in which the surface areas of the aluminium parts are visible. The failure models are marked in Figure 19b with the letters “M” for mixed model and “C” for cohesive model.

Expressing the results in stress, the range for the five tested batches is between 0.95 MPa and 1.57 MPa. These are relatively small values, but it must be remembered that the composite substrate is responsible for carrying the load, while the coating performs other functions such as erosion protection, impact protection and aesthetics. The main factor to which the coating is exposed in terms of strength is wind suction, for example, when using this type of coating in façade panels. In the literature [49], we can find information that adhesion at 0.3 MPa is considered “forceful”. Hence, these types of new products can be used in civil engineering.

### 3.3. 3-PB Tests of PC “Sandwich” Composites

**Figure 20:**
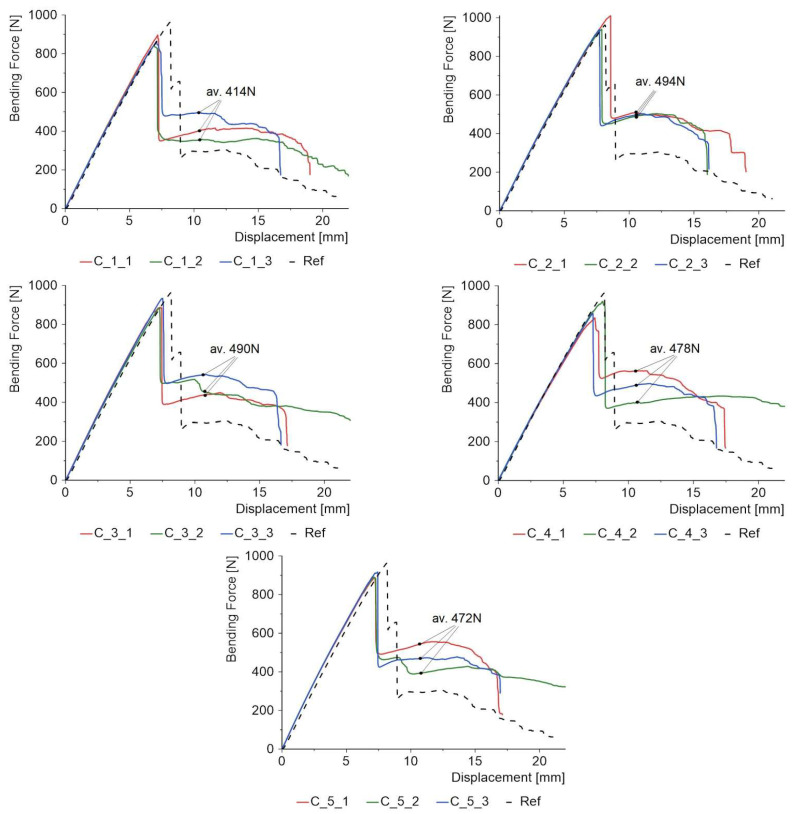
Force–displacement diagrams for 3-PB specimens.

The proposed PCs could have a number of applications, including coatings on façade panels, bridge girders or water sports equipment; hence, it is necessary to determine how the applied coating influences bending strength, absorbed energy and the failure process. 3-PB tests were carried out on 120 mm × 30 mm beams with a support centreline spacing of 100 mm. The coating was on the side of the tension zone (Figure 10). Figure 20 collects the force–displacement plots for the three specimens in each batch. The dotted line shows the mean result for the uncoated reference specimens. The maximum force for the reference specimens is 962 N, so almost every batch with a coating obtained a lower or the same value. The worst result is shown by batch C_1 with silicon carbide powder; in this case, the strength drop is 9.77%. Thus, considering the maximum value of the bending force, the coatings contribute to a slight reduction in strength. However, it should be remembered that these types of coatings are mainly intended to play a functional and decorative role.

However, the benefits of PC become clearer when we have a closer look at the force diagrams after the maximum value is reached. For the reference specimens, the force drops to a level of 295 N, or about 69%. For the coated specimens, the percentage drops are in the range of 45% to 52%, which means that the additional coating has a very positive effect on the strength at failure after the maximum force is reached. This phenomenon requires further research, but it is possible to formulate the hypothesis that the additional layer introduces a disproportion between the stresses in the upper and lower laminate layers. Hence, initially, one of the laminates is damaged, while the other remains integral and can carry about half the load, with the average values for three specimens being shown in Figure 20.

**Figure 21:**
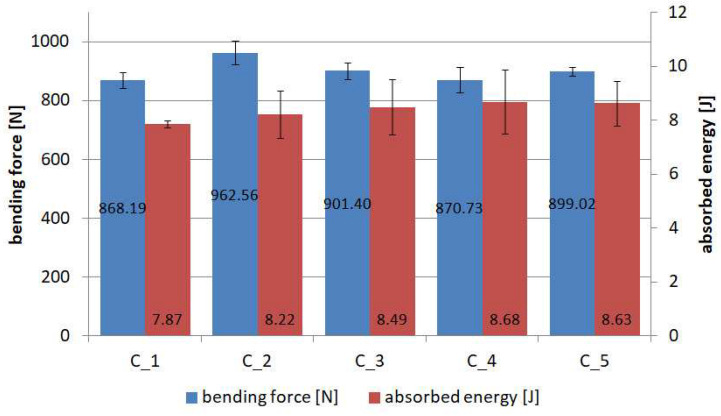
Summary of average maximum forces and absorbed energy during 3-PB tests.

Figure 21 summarises the values of both forces and absorbed energy calculated as the area under the entire force–displacement diagram. The average absorbed energy for the reference specimens without coating was 7.153 J, so a higher value for this parameter is obtained in each batch after coating is applied.

The percentage increase in absorbed energy after application of the coating compared to the reference specimens is in the range of 10% to 20.65%. Thus, this type of structure becomes safer considering the entire range of material damages. The average percentage standard deviation for force is on the low level in the range of 1.75% to 5.04%, while for absorbed energy, it is in the range of 1.73% to 13.71%.

**Figure 22:**
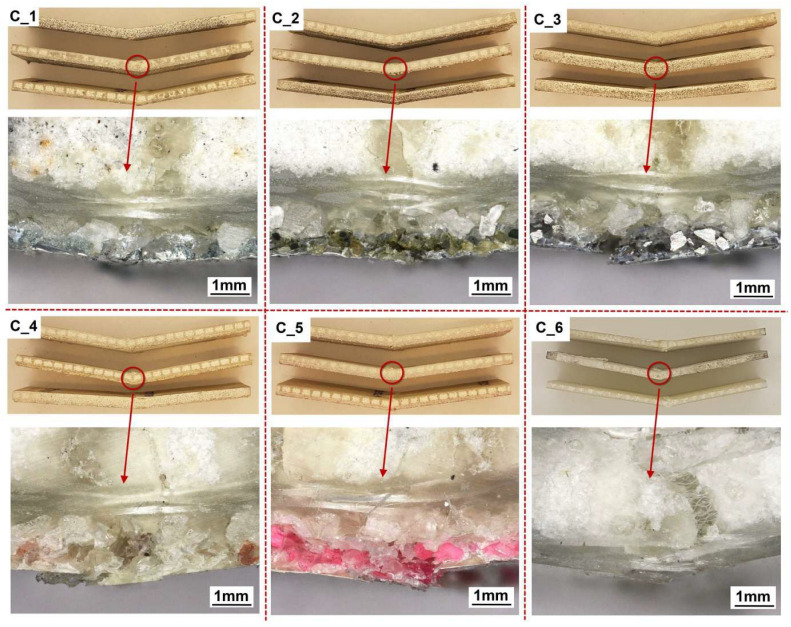
View of specimens after 3-PB tests.

The specimens are still integral after three-point bending tests (Figure 22). This is due to the fact that the core of the composite fails at higher strains. Furthermore, in the laminate itself, the stresses change along the thickness, and the fibre strands closer to the core also maintain integrity. Most significantly, there is no delamination of the PC from the composite substrate. In the zones shown in half of the specimens in Figure 22, only small cracks in the FL region are visible under the microscope. The electro-corundum grains of the BL, which are partially bonded to the polyester matrix, remain integral to the laminate surface. For the coated specimens, by observing the lower fibre strands of the laminate, no cracks are visible, and this is due to the additional alumina layer of the PC BL. The "Soric" core is also not cracked. The situation is different for the reference sample. Large deformations and cracking in the core are visible, as well as a damaged laminate layer.

In summary, PCs do not contribute to increasing the maximum bending force of the “sandwich” composite but allow it to work more safely after the first failure is reached. They absorb more energy and protect the laminate and core even under large deformations.

## 4. Conclusions

The technology presented in this work makes it possible to manufacture new products such as pre-impregnated coatings in a controlled, reproducible and efficient manner. The PCs obtained are self-contained products with high flexibility, which enables their use also on curved surfaces. The construction and functionality of the original machine for the semi-automatic production of PCs is also presented. The step-by-step manufacture of PC and its subsequent application as a coating of a “sandwich” composite was discussed. The work presents their use in vacuum infusion technology, but PC can also be applied using other techniques such as hand lamination, autoclave curing or winding. This article shows that PC coatings are durable in terms of mechanical loads in the field of adhesion and bending. This fact, combined with the presented manufacturing technology, which offers extensive possibilities in the design of PC, means that there is great potential for improving the durability of FRP composites. PC coatings can, for example, be a carrier for flame-retardant materials while maintaining high aesthetics. This paper also shows that it is possible to create PC layers from grains with a wide range of gradations. This means that, depending on the loads, e.g., erosion, concentrated static and dynamic loads, the thickness of the coating can be adjusted to specific requirements.

The following conclusions can be drawn from the work carried out:

- The choice of gradation of the FL and the BL, as well as the proportion of thermoplastic material, has a significant impact on the final thickness and grammage of the PC. For the materials used in this work, PC thicknesses in the range 1.11 to 1.41 mm and grammages from 1.37 to 2.35 kg/m^2^ were obtained;
- Considering the failure force of a 30 mm wide PC, it is in the range of 24.61 N to 28.73 N. This is an important parameter from a technological point of view, e.g., when using winding where material tension must be ensured;
- The Young’s modulus, depending on the FL powders used, can vary between 38.58 MPa and 84.15 MPa, and the Poisson’s ratio varies from 0.26 to 0.34. This is important information that can be used to build a global numerical model in the future;
- The PC on the “sandwich” composite has satisfactory adhesion ranging from 0.95 MPa to 1.57 MPa, which allows it to be used in civil engineering;
- Three-point bending tests have shown that the one-sided application of PC results in a decrease in maximum force of approximately 9.77% compared to reference specimens without PC. This result was achieved for the C_1 batch with silicon carbide as the FL. The large advantages of using PC appear at the bending stage after the maximum force has been reached. PC makes the absorbed energy higher, and also the force drops by about 45% to 52% from the maximum value. For reference specimens, the decrease in force after the maximum value is as high as 69%;
- Pre-impregnated coatings made of powders with a variety of colours highlight the wide decorative possibilities of the products. PCs, therefore, have a high implementation potential.

Directions for further work include the use of the BL in the form of synthetic or natural fibre fabrics, the use of multi-powder FL, the construction of more precise powder feeders and the continuation of tests not only under static but also dynamic loads, as well as testing the effects of high temperatures and environmental loads.

## Figures and Tables

**Table 1:** Powder parameters.

Batch Number	Batch 1	Batch 2	Batch 3	Batch 4	Batch 5	Batch 1–5
Type of FL powder	silicon carbide	glauconite	stainless-steel shot	quartz sand	chromium electro-corundum	white electro-corundum
FEPA-F granularity	F220	F60	F050	F080	F036	F24
Grain size [μm]	58	260	336	185	525	745
Density of powder material [g/cm^3^]	3.21	2.5	7.8	2.65	3.95	3.95

**Table 2:** Parameters of manufactured PCs.

Batch Number	Batch 1	Batch 2	Batch 3	Batch 4	Batch 5	Batch 6
Type of FL powder	silicon carbide	glauconite	stainless-steel shot	quartz sand	chromium electro-corundum	-
PC grammage [kg/m^2^]	1.37	1.62	2.35	1.97	1.98	0.25
PC grammage [kg/m^2^]	1.11 ± 0.08	1.19 ± 0.06	1.27 ± 0.06	1.11 ± 0.06	1.41 ± 0.05	0.29 ± 0.02
Percentage of non-woven	18.36	15.56	10.72	12.79	12.70	100

**Table 3:** Thicknesses of the obtained “sandwich” composites with PC.

	Silicon Carbide	Glauconite	Stainless Steel Shot	Quartz Sand	Chromium Electro-Corundum	Ref. (Without PC)
thickness [mm]	5.26 ± 0.07	5.46 ± 0.06	5.66 ± 0.05	5.69 ± 0.09	5.77 ± 0.05	4.12 ± 0.03

**Table 4:** Coefficients of a polynomial function.

	Batch 1	Batch 2	Batch 3	Batch 4	Batch 5	Batch 6
a_1_ [kN/mm]	15.26	19.5	14.42	27.88	14.62	3.08
a_2_ [kN/mm^2^]	−3.50	−4.6	−2.58	−13.40	−2.79	−0.0738
a_3_ [kN/mm^3^]	0.217	0.358	0.145	3.19	0.157	-
a_4_ [kN/mm^4^]	-	-	-	−0.297	-	-
coef. of det.	0.999	0.999	1.000	0.998	0.999	1.000

## Data Availability

The original contributions presented in this study are included in the article. Further inquiries can be directed to the corresponding author.

## Abbreviations

The following abbreviations are used in this manuscript:

FRP	fibre-reinforced polymer
PC	pre-impregnated coating
FL	functional layer
BL	backing layer
3-PB	three-point bending
DIC	digital image correlation
GFRP	glass fibre-reinforced polymer
CFRP	carbon fibre-reinforced polymer
UV	ultraviolet
UHMWPE	ultra-high-molecular-weight polyethylene
7YSZ	7% yttria-stabilised zirconia
APS	atmospheric plasma spray
TBC	thermal barrier coating
PVA	poly(vinyl alcohol)
APP	ammonium polyphosphate
SP	sepiolite nanofillers
PMC	polymer matrix composite
SEM	scanning electron microscope
MEKP	methyl ethyl ketone peroxides
%RSD	percent relative standard deviation
